# Transcriptional and neurotransmitter signatures associated with regional gray matter alterations in juvenile myoclonic epilepsy

**DOI:** 10.3389/fnmol.2026.1693722

**Published:** 2026-01-29

**Authors:** Xiao-Yi Liu, Han Xu, Si-Yu Gu, Hu-Cheng Yang, Chuan-Xu Luo, Shu Wang, Wen-Hui Li, Ping-Lei Pan

**Affiliations:** 1 Department of Radiology, Affiliated Hospital 6 of Nantong University, Yancheng Third People’s Hospital, Yancheng, China; 2 Binhai Maternal and Child Health Hospital, Yancheng, China; 3 Yancheng Maternal and Child Health Care Hospital, Yancheng, China; 4 Department of Neurology, Affiliated Hospital 6 of Nantong University, Yancheng Third People’s Hospital, Yancheng, China

**Keywords:** gray matter, juvenile myoclonic epilepsy, neurotransmitter systems, transcriptomics, voxel-based morphometry

## Abstract

**Introduction:**

The neurobiological basis of gray matter (GM) alterations in juvenile myoclonic epilepsy (JME) remains poorly understood. This study aimed to identify robust GM changes and their underlying molecular signatures.

**Methods:**

We performed an updated coordinate-based meta-analysis of 15 voxel-based morphometry studies (394 JME patients, 448 healthy controls) and integrated the resulting GM alteration map with data from the Allen Human Brain Atlas and neurotransmitter atlases using advanced spatial correlation, gene enrichment, and network analysis approaches.

**Results:**

Our analysis revealed a consistent pattern of GM decreases in the sensorimotor areas and increases in regions implicated in emotion and cognition. These structural changes were spatially correlated with a set of 926 genes enriched for pathways related to ion channel activity, synaptic function, neuronal processes, and cellular metabolism, which showed peak expression during neurodevelopmental periods coinciding with JME onset. Protein–protein interaction analysis identified hub genes from two key functional classes: transcriptional regulators linked to circadian rhythms, and cellular signaling molecules including established monogenic epilepsy genes. Furthermore, the GM map correlated significantly with the spatial distributions of the serotonin, dopamine, and acetylcholine neurotransmitter systems.

**Discussion:**

While these associations are based on data from healthy donors and require further validation, our findings bridge the gap between macroscopic brain alterations and their underlying molecular architecture in JME. This provides an integrated model of its pathophysiology and highlights potential therapeutic avenues.

## Introduction

Juvenile myoclonic epilepsy (JME) is a prevalent subtype of generalized epilepsy, typically manifesting during adolescence (Rubboli et al., 2023; Chen et al., 2020). The condition is characterized by a distinctive triad of myoclonic jerks, generalized tonic–clonic seizures, and occasional absence seizures (Hirsch et al., 2022). With an estimated 1–2 individuals per 1,000 globally, JME accounts for approximately 8.7% of all epilepsy cases (Asadi-Pooya et al., 2015; Berg and Scheffer, 2011; Syvertsen et al., 2017). The lifelong nature of JME is marked by significant management challenges, including treatment-refractory seizures and a high propensity for relapse upon medication withdrawal, often complicated by psychiatric comorbidities that adversely affect patient outcomes (Serafini et al., 2019; Pietrafusa et al., 2021). Current research indicates that JME has a significant genetic component, supported by findings of familial aggregation and the identification of genes such as GABRA1, EFHC1, and BRD2 (Santos et al., 2017; Martínez-Juárez et al., 2006; de Nijs et al., 2009; Peljto et al., 2014). Furthermore, its pathophysiology has been associated with neurotransmitter system dysregulation, particularly in the dopaminergic system (Loucks et al., 2019; Ciumas et al., 2008). However, despite these advances, a comprehensive understanding of the underlying mechanisms driving JME remains elusive (Zhong et al., 2018).

Numerous neuroimaging studies have revealed structural brain alterations in patients with JME, significantly advancing our understanding of the disorder’s neural basis (Zhong et al., 2018; Luo et al., 2023; Ke et al., 2024). Among these, voxel-based morphometry (VBM) (Ashburner and Friston, 2000; Zhang et al., 2022; Knake et al., 2017) has been especially widely utilized to detect regional gray matter (GM) volume or density changes in patients with JME compared to healthy controls (HCs), frequently implicating regions such as the thalamus, frontal gyrus, postcentral gyrus, putamen, hippocampus, and insula (Ke et al., 2024; Betting et al., 2006; Woo et al., 2006; De Araújo Filho et al., 2009). However, a persistent challenge in this field is the considerable heterogeneity among studies, reflected in inconsistencies regarding the precise location, extent, and even the direction (increase or decrease) of reported GM alterations (Roebling et al., 2009; Swartz et al., 2016; O’Muircheartaigh et al., 2011). This variability is likely attributable to factors such as small sample sizes, clinical differences across patient cohorts (e.g., age, medication use, seizure characteristics), and methodological differences in imaging acquisition and analysis protocols (Zhu et al., 2022).

To address these limitations and extract robust patterns from a heterogeneous literature, coordinate-based meta-analysis (CBMA) has emerged as an essential approach in neuroimaging research. By aggregating coordinates from multiple independent studies, CBMA enhances statistical power and increases the ability to detect consistent and reliable patterns of GM alterations specifically associated with JME (Tench et al., 2020; Salimi-Khorshidi et al., 2009). For example, a preliminary CBMA of 12 VBM studies conducted by Kazis and colleagues identified significant GM increase, notably in the left precentral gyrus, right superior frontal gyrus, left median cingulate/paracingulate gyri, and supplementary motor areas bilaterally, and GM decrease in the left thalamus and left insula (Kazis et al., 2021). Despite these important advances, CBMA primarily reveals convergent neuroanatomical changes, whereas the underlying molecular mechanisms remain largely unexplored. Bridging these macroscopic brain alterations with their molecular underpinnings is critical for advancing our pathophysiological understanding of JME.

Given the strong heritability of JME (Santos et al., 2017; Delgado-Escueta, 2007; Pal et al., 2003; Elmslie et al., 1997; Guaranha et al., 2011), investigating the transcriptional signatures associated with regions of convergent GM alteration represents a promising avenue. This strategy, known as “imaging transcriptomics,” integrates neuroimaging results with spatial brain-wide gene expression data, such as that provided by the Allen Human Brain Atlas (AHBA), to uncover the molecular architecture underlying brain structure and function (Arnatkeviciute et al., 2023; Hawrylycz et al., 2012). In parallel, neurotransmitter systems play critical roles in the pathogenesis of JME (Hattingen et al., 2014; Esmail et al., 2015). Nuclear imaging techniques such as single-photon emission computed tomography (SPECT) and positron emission tomography (PET) enable *in vivo* mapping of neurotransmitter receptor distributions across the human brain (Beliveau et al., 2017; Lehto et al., 2015). These maps can be linked to anatomical brain structures (Krampfl et al., 2005; Meschaks et al., 2005) via the JuSpace toolbox (Dukart et al., 2021). This can yield novel insights into whether structural abnormalities co-localize with specific neurotransmitter systems. Although approaches linking brain structural changes to gene expression (Cai et al., 2022) or neurotransmitter maps (Li et al., 2024; Premi et al., 2023) have provided valuable understanding in other neuropsychiatric conditions, no study has yet combined both transcriptomic and neurochemical mapping with meta-analytic GM findings in JME.

**Figure 1:**
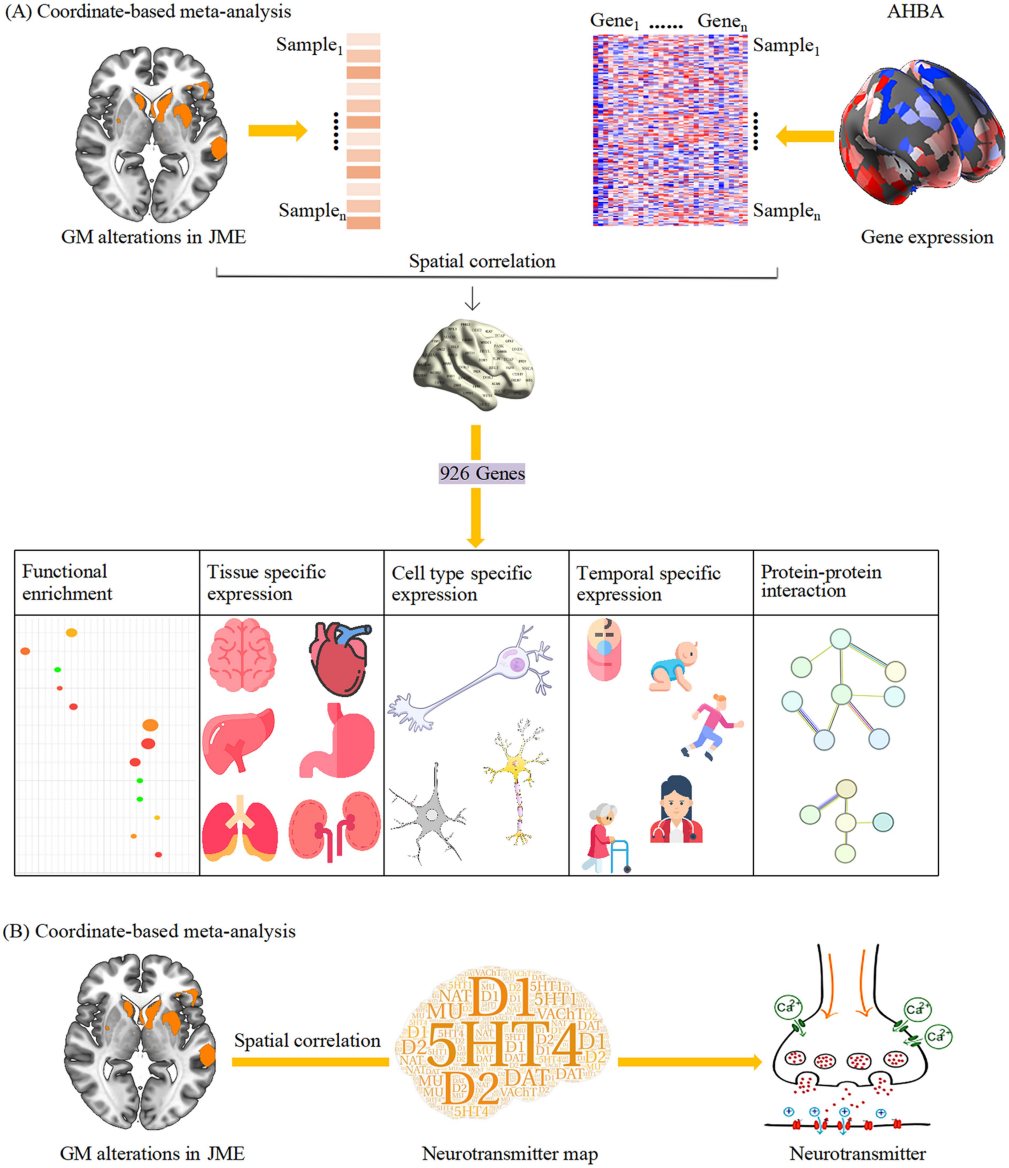
A systematic flowchart of the study design. **(A)** The results of CBMA were linked to gene expression data. Enrichment analyses were performed subsequently on the overlapped genes. **(B)** In a parallel analysis, the JME-associated GM alteration map was spatially correlated with neurotransmitter receptor and transporter atlases. AHBA, Allen Human Brain Atlas; CBMA, coordinate-based meta-analysis; GM, gray matter; JME, juvenile myoclonic epilepsy.

In recent years, an increasing number of VBM studies have become available (Ke et al., 2024; Zhang et al., 2022), warranting an updated CBMA to improve the reliability of structural findings in JME. Accordingly, the present study aimed to combine an updated CBMA of GM alterations in JME with imaging transcriptomic and neurotransmitter receptor mapping, to elucidate the molecular and neurochemical signatures underlying these structural changes. This integrated multi-modal approach may shed new light on the complex interplay between brain structures, genetic expression, and neurochemical systems in the pathophysiology of JME. A systematic flowchart of the study design is presented in Figure 1.

## Materials and methods

### Literature search and selection

A comprehensive systematic search was performed across three electronic databases (PubMed, Web of Science, and Embase) to identify studies published up to December 13, 2024. To minimize time-lag bias, we updated the search on May 20, 2025, using the same strategy. This update yielded no new eligible publications. We identified suitable studies by searching with the key terms detailed subsequently: “juvenile myoclonic epilepsy” OR “JME” AND “VBM” OR “morphometry” OR “voxel-based morphometry” OR “gray matter” OR “grey matter.” Additionally, the search was expanded by manually scrutinizing the reference sections of all selected articles and any relevant CBMA/review literature encountered to identify further eligible studies. Only studies that directly compared GM alterations between patients with JME and HCs were included. The study selection flow is detailed in Supplementary Figure S1.

Studies were required to meet the following criteria for inclusion: (1) representing original research disseminated as an article in an English-language, peer-reviewed journal; (2) employed a VBM approach to compare regional GM differences between patients with JME and HCs; (3) conducted VBM analysis at the whole-brain level rather than only region-of-interest analysis; (4) documented findings using coordinates in standardized Montreal Neurological Institute (MNI) or Talairach stereotactic space; (5) included patients with JME without other significant concurrent medical, neurological, or psychiatric disorders. For studies with longitudinal data, only baseline comparisons were considered. The exclusion criteria were: (1) non-original studies (e.g., reviews, meta-analyses, meeting abstracts, letters, and case reports); (2) involved animal models; (3) did not include a comparison between patients with JME and HCs; (4) included small sample size with seven or fewer individuals either in the JME or HCs group; (5) did not report results as peak coordinates in MNI or Talairach space; (6) involved data suspected of overlapping with other included publications. To ensure a clear and detailed description of our research methods and results, we adhered to relevant guidelines and recommendations for literature selection (Shamseer et al., 2015).

The collected data from the analyzed studies encompassed: first author, publication date, the total number of participants, gender distribution, mean age, illness duration, magnetic field strength, software for image analysis, adjusted variables, significance threshold, peak metrics reported as *t*-values, *z*-values, or *p*-values, with conversion tools for *z* and *p* to *t* available at (SDM Statistics),^1^ and the coordinate system used for brain mapping (MNI or Talairach).

### Voxel-wise CBMA

A CBMA was conducted to examine regional GM alterations between patients with JME and HCs, employing the anisotropic effect size version of signed differential mapping (AES-SDM) software.^2^ A comprehensive explanation of the SDM techniques can be found within the AES-SDM tutorial^3^ and related scholarly articles (Radua et al., 2012; Radua et al., 2014). Initially, we constructed a database including sample characteristics, peak activation coordinates, effect size estimates, and key clinical parameters (notably mean age and duration of illness in the JME cohort). Subsequently, a pooled analysis was conducted to examine the GM changes between patients with JME and HCs. The standard AES-SDM kernel size and thresholds were applied [full width at half maximum (FWHM) = 20 mm, voxel *p* < 0.005, peak height *z* = 1, and cluster extent = 50 voxels], which achieve an effective equilibrium between false positive and false negative findings. Ultimately, a resultant z-map, visualized via MRIcron^4^ to depict GM alterations, was generated through a voxel-by-voxel random-effects averaging of the GM difference maps from the contributing studies. The computational approach accounted for factors including sample sizes, variance within individual studies, and the heterogeneity observed across studies.

Several analyses were employed to confirm the robustness and reliability of our CBMA findings. To investigate unexplained variability across studies, an analysis of inter-study heterogeneity was conducted. Significant heterogeneity was indicated by voxels meeting a *p*-value < 0.005, a peak *z*-score of 1, and forming a cluster of at least 10 voxels. To assess the robustness of the main meta-analytic findings, we employed a jackknife sensitivity procedure. This involved iteratively removing each study in turn and then re-calculating the meta-analysis. Potential publication bias was investigated using Egger’s tests (Irwig et al., 1998); a *p*-value under 0.05 was interpreted as signifying notable bias. Furthermore, funnel plots were generated to visually inspect for bias, with marked asymmetry alongside a *p*-value < 0.005 suggesting its presence.

### Brain gene expression data processing

For our analysis, brain gene expression information was sourced from the AHBA dataset (Hawrylycz et al., 2012; Hawrylycz et al., 2015).^5^ The foundation of this dataset is tissue gathered from six human individuals post-mortem (Supplementary Table S1). Gene expression levels for over 20,000 genes were quantified in 3702 spatially discrete brain tissue samples using custom 64 K Agilent microarrays. A recently described pipeline was implemented for processing the resulting gene expression data (Arnatkeviciute et al., 2019). Specifically, the initial procedural step was to refresh the probe-to-gene annotations. This was achieved through the use of the Re-Annotator package, which integrated the most current information obtained from the National Center for Biotechnology Information (Arloth et al., 2015). An intensity-based filtering step was applied, removing probes whose signal intensity did not surpass the background noise threshold in at least 50% of the samples across the entire donor cohort. Furthermore, considering that expression levels for individual genes were often measured by multiple probes, RNA Sequencing (RNA-seq) data were utilized as a reference standard to guide the selection of the most appropriate probe for each gene. After filtering out genes not common to both RNA-seq and microarray datasets, we calculated the correlation between the two expression measures for the remaining genes. Probes were then filtered based on correlation strength, excluding those with *r* < 0.2 compared to RNA-seq data. When multiple probes corresponded to a single gene, the probe exhibiting the strongest correlation with RNA-seq data was designated as the gene’s representative. A noteworthy constraint in data availability was observed: while all six donors contributed left hemispheric data, information for the right hemisphere was restricted to only two of these individuals. Moreover, potential bias could arise from including subcortical samples, as gene expression differs markedly between cortical and subcortical regions (Hawrylycz et al., 2012). Normalization procedures were implemented to control for potential discrepancies between samples and donor-specific gene expression effects. This involved applying the scaled robust sigmoid normalization method for both within-sample (cross-gene) and within-gene (cross-sample) adjustments. Our approach further incorporated the principle of differential stability (DS). DS quantifies the inter-individual consistency of regional gene expression. Previous research suggests that genes exhibiting high DS display reproducible spatial profiles across donors and are enriched in brain-related biological functions (Hawrylycz et al., 2015). We ranked the genes based on their DS values and retained the top 50% for the main analysis, as gene expression conservation across subjects is a fundamental requirement for transcriptome-neuroimaging spatial correlations (Zhao et al., 2023; Liu et al., 2022). These processing procedures culminated in a dataset containing normalized expression data for 5,013 genes derived from 1,290 tissue samples. To ensure consistency with the neuroimaging CBMA, our analyses were confined to the GM mask supplied by the AES-SDM. This process yielded a final sample-by-gene matrix with dimensions of 926 × 5,013. To validate the robustness of our findings against potential variations in data processing, we performed sensitivity analyses. While the primary analysis focused on the top 50% of genes with the highest DS values to ensure conserved expression patterns across the six donors, we also replicated the analysis using alternative thresholds (top 40 and 60%) to assess the impact of cutoff selection.

### Gene category enrichment analysis

To probe the genetic foundations associated with GM alterations of JME, we utilized transcriptome-neuroimaging spatial correlation analysis combined with the recently introduced ensemble-based gene category enrichment analysis (GCEA). For the spatial correlation component, we first defined a spherical region of interest (3 mm radius) centered on the MNI coordinates of each individual brain tissue sample. Within each sphere, the average *t*-value was extracted from the statistical map representing correlations between GM alterations and JME. Subsequently, Pearson correlations were computed for each gene individually, comparing its expression profile across tissue samples with the corresponding average *t*-values. This procedure generated 5,013 spatial correlation coefficients, which are hereafter designated as “gene scores.” Following a methodology consistent with that of Fulcher et al. (2021), the gene scores were then subjected to neuroimaging-spatial ensemble-based GCEA. This analysis began by obtaining the latest gene ontology (GO) term hierarchy and annotation data from the GO portal.^6^ Second, the next procedural step involved directly annotating the 5,013 AHBA genes to specific GO categories. We then limited our subsequent analytical work to consider only those GO categories that contained a gene annotation count ranging from 10 to 200. Subsequently, gene scores were aggregated for each GO category; this involved computing the average score of all genes assigned to a particular category. Following this, 10,000 surrogate maps were created employing the brainsmash package.^7^ These maps utilized a spatial-lag model to ensure their spatial autocorrelation structure mirrored that of the original *t*-map (Burt et al., 2020). Null distributions representing the expected mean gene scores for each GO category under spatial constraints were generated. This involved calculating spatial correlations between the gene expression data and the 10,000 surrogate maps designed to preserve spatial autocorrelation (forming a neuroimaging-spatial ensemble-based null model). Ultimately, the statistical significance of each GO category was determined by comparing its score derived from the actual empirical data against this neuroimaging-spatial ensemble-based null distribution. A two-sided significance threshold of *p* < 0.05 was applied, identifying categories with scores significantly higher or lower than anticipated by the null model.

### TSEA, CSEA and SEA

We employed the online tissue specific expression analysis (TSEA) tool^8^ and cell type specific expression analysis (CSEA) tool^9^ (Dougherty et al., 2010) to investigate whether the identified genes associated with JME demonstrated enriched expression within particular tissues, cell types, or specific temporal developmental periods. The likelihood of gene expression specificity was categorized using the specificity index probability (pSI values of 0.05) (Xu et al., 2014). The statistical significance of the enrichment analysis was evaluated using Fisher’s exact test, with *p*-values adjusted for multiple testing via false discovery rate (FDR) by the Benjamini-Hochberg correction method, setting the significance threshold at a corrected *p*-value < 0.05.

### PPI analysis

Protein–protein interaction (PPI) analyses were performed using STRING v11.0^10^ to construct interaction networks. In addition, the networks from the STRING database are displayed in the main Cytoscape window and potential hub genes within the set of JME-related genes were identified. Hub genes were characterized as the top 10% of nodes ranked by connectivity (node degree), considering only interactions meeting a minimum confidence score of 0.9.

### Correlation with neurotransmitters

To link JME-related GM alterations to specific neurochemical systems, we utilized the JuSpace toolbox.^11^ Spatial correlations (Pearson’s *r*) were computed between the meta-analytic *t*-map and PET/SPECT maps for nine neurotransmitter systems (e.g., dopamine, serotonin) (Hansen et al., 2022). These correlations corrected for spatial autocorrelation, with significance determined via 5,000 spatial permutations (*p* < 0.01, FDR corrected). To ensure the robustness of these results, we further applied a strict Bonferroni correction.

## Results

### Included studies and sample characteristics

Sixteen studies initially met the inclusion criteria (Zhong et al., 2018; Ke et al., 2024; Zhang et al., 2022; Knake et al., 2017; Betting et al., 2006; Woo et al., 2006; de Araújo Filho et al., 2009; Roebling et al., 2009; Swartz et al., 2016; O’Muircheartaigh et al., 2011; Kim et al., 2007; Woermann et al., 1999; Ciumas et al., 2010; Liu et al., 2011; Öztürk et al., 2020; Saini et al., 2013). However, as the participant sample of one study (de Araújo Filho et al., 2009) overlapped with that of another (Betting et al., 2006), it was excluded from the current CBMA. Finally, 15 studies with a total of 394 patients with JME and 448 HCs (Zhong et al., 2018; Ke et al., 2024; Zhang et al., 2022; Knake et al., 2017; Betting et al., 2006; Woo et al., 2006; Roebling et al., 2009; Swartz et al., 2016; O’Muircheartaigh et al., 2011; Kim et al., 2007; Woermann et al., 1999; Ciumas et al., 2010; Liu et al., 2011; Öztürk et al., 2020; Saini et al., 2013) were selected for the CBMA, featuring a mean participant count of 26.3 per study (range, 13–67). The average age for JME across studies was approximately 25.8 years, and 235 of these participants were female. Similarly, 448 HCs were included, with a mean of 29.9 participants per study. Data on disease duration were provided by 10 of the 15 datasets (range, 6.1–40.4 months). Magnetic resonance imaging (MRI) data acquisition involved 1.5 T scanners (8 studies) and 3.0 T scanners (6 studies), while one study utilized a 2.0 T scanner. Across the included datasets, neuroimaging data analysis was predominantly performed using statistical parametric mapping (SPM), in its various iterations (SPM96, SPM2, SPM5, SPM8, SPM12). This software was employed in 14 datasets, with the remaining study utilizing FMRIB software library (FSL). Comprehensive demographic, clinical, and imaging details are presented in Supplementary Table S2.

### GM alterations in JME

**Figure 2:**
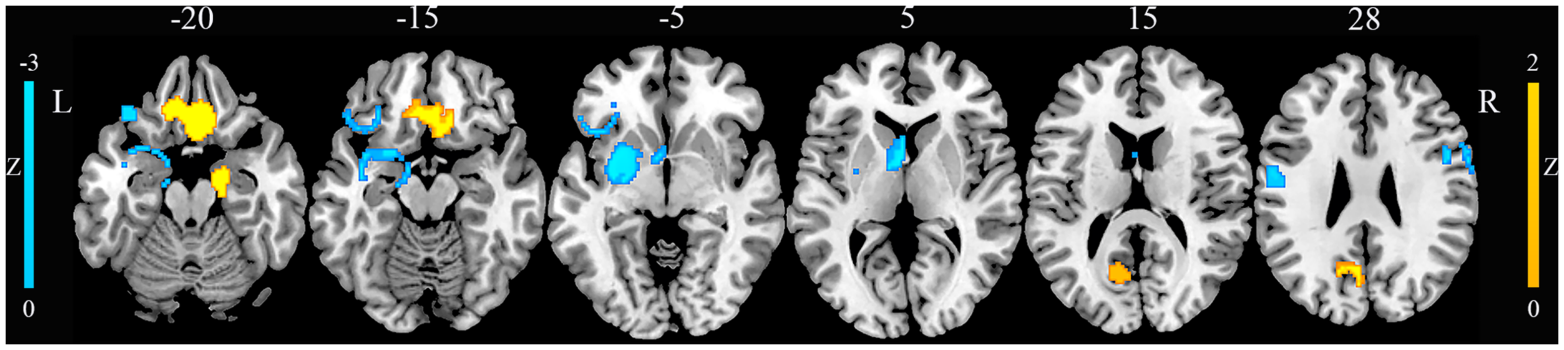
Gray matter alterations in juvenile myoclonic epilepsy. This figure illustrates brain regions showing significant gray matter alterations between patients with juvenile myoclonic epilepsy and healthy controls. L, left; R, right.

Compared with HCs, patients with JME exhibited increased GM in the right gyrus rectus, right parahippocampal gyrus, and left precuneus (Figure 2). Conversely, patients with JME showed decreased GM in the left putamen, left postcentral gyrus, right precentral gyrus, and left inferior frontal gyrus (Figure 2). The jackknife sensitivity analyses reveled that the above-mentioned regional GM alterations were highly robust in JME. The heterogeneity analysis revealed significant unexplained between-study variability of GM changes in brain regions, including the left inferior frontal gyrus and left precuneus. The funnel plots showed no obvious asymmetric of all significant brain regions. The quantitative assessment measured by Egger’s tests revealed publication bias in the right parahippocampal gyrus (Table 1).

**Table 1:** Gray matter alterations in juvenile myoclonic epilepsy identified by CBMA.

Brain regions	Peak MNI coordinates (x, y, z)	Number of voxels	SDM-Z value	*p*	Heterogeneity	Egger’s test (*p*)	Jackknife sensitivity analysis
Juvenile myoclonic epilepsy > healthy controls							
Right rectus gyrus	6, 20, −26	776	1.565	0.00076	No	0.78	15/15
Right parahippocampal gyrus	22, −2, −32	408	1.524	0.00097	No	0.019	15/15
Left precuneus	-14, −60, 20	199	1.521	0.00099	Yes	0.059	13/15
Juvenile myoclonic epilepsy < healthy controls							
Left putamen	−32, −6, −4	577	−2.195	0.000031	No	0.23	15/15
Left postcentral gyrus	−48, −10, 30	197	−1.914	0.00071	No	0.16	14/15
Left inferior gyrus	−32, 26, −8	158	−1.922	0.00066	Yes	0.050	13/15
Right precentral gyrus	48, 4, 30	145	−1.916	0.00069	No	0.058	13/15

CBMA, coordinate-based meta-analysis; MNI, Montreal Neurological Institute; SDM, signed differential mapping.

### Gene category enrichment analysis

**Figure 3:**
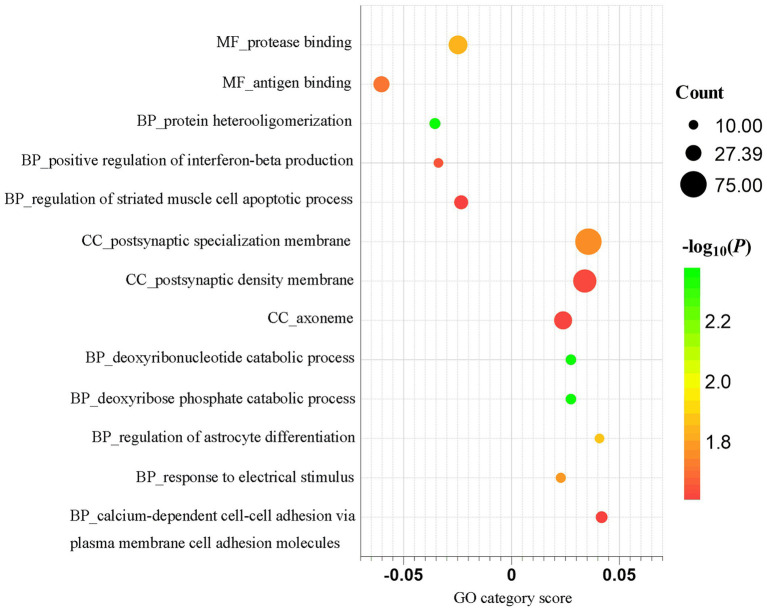
Functional enrichment analysis of genes associated with gray matter alterations in juvenile myoclonic epilepsy. The *x*-axis represents category scores, while the *y*-axis lists GO terms. Bubble size is proportional to the number of overlapping genes. Bubble color indicates the statistical significance of spatial correlations, calculated via spatially constrained permutation and presented as -log_10_(*P*). GO, gene ontology.

Functional enrichment analysis of the identified GM-associated genes revealed significant GO terms. The results are detailed in Figure 3. Genes whose expression was positively correlated with regional GM were enriched for terms such as deoxyribonucleotide catabolic process, deoxyribose phosphate catabolic process, regulation of astrocyte differentiation, response to electrical stimulus, calcium-dependent cell–cell adhesion via plasma membrane cell adhesion molecules, postsynaptic specialization membrane, postsynaptic density membrane and axoneme. Genes whose expression was negatively correlated with regional GM were enriched for terms including protein heterooligomerization, positive regulation of interferon-beta production, regulation of striated muscle cell apoptotic process, protease binding and antigen binding (Supplementary Table S3). To evaluate the impact of threshold selection, we performed a sensitivity analysis using alternative DS cutoffs (top 40 and 60%), which yielded normalized datasets of 4,010 and 6,016 genes, respectively. After repeating the full analytical procedure on these datasets, we found that the analysis using the top 60% gene set, in particular, revealed that the identified GO categories exhibited substantial overlap with those from our main analysis. The complete results for the 40 and 60% thresholds are presented in Supplementary Tables S4, S5.

### Tissue-, cell type-, and temporal-specific expression

**Figure 4:**
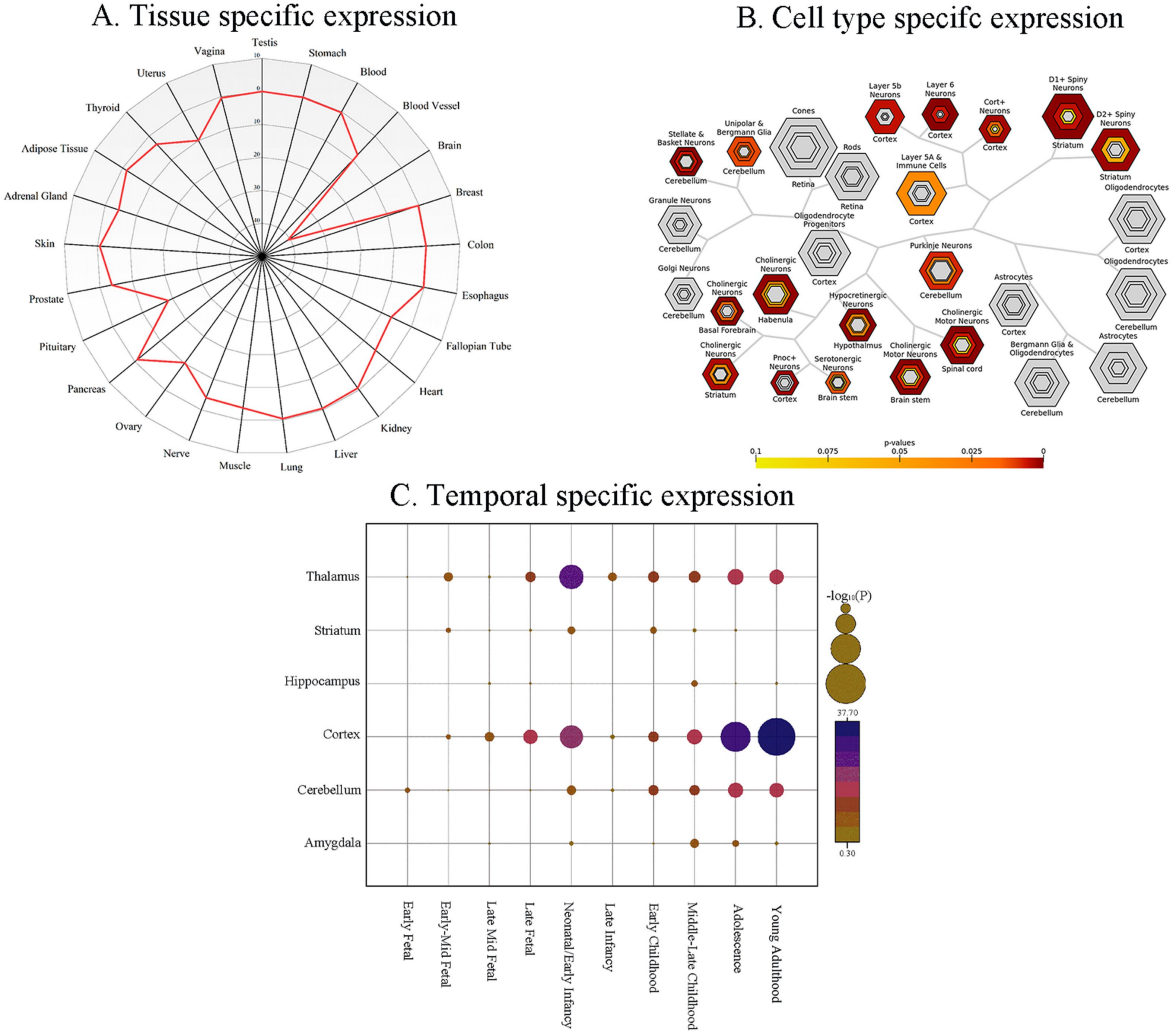
Specific expression of the genes with gray matter alterations in juvenile myoclonic epilepsy. Significance is determined by *p*-values corrected using FDR-BH method (threshold *p* < 0.05). **(A)** Tissue specific expression analysis. The radar chart illustrates gene expression enrichment across various human tissues. The radial axis indicates the significance of enrichment, expressed as −log_10_(*p*-value), for each tissue. **(B)** Cell type specific expression analysis. Hierarchical clustering of cell types based on transcript levels of the analyzed genes, highlighting preferential expression patterns. **(C)** Temporal expression analysis. The *x*-axis denotes developmental stages of the human brain, and the *y*-axis denotes distinct brain regions. Bubble size is proportional to the −log_10_(*p*-value), indicating the significance of expression specificity for each developmental stage and brain region. FDR-BH, Benjamini-Hochberg false discovery rate.

Gene set enrichment and specificity analyses revealed significant findings across tissue, cell type, and developmental time scales. TSEA demonstrated significant enrichment in both brain tissue and the pituitary gland (Figure 4A). CSEA further pinpointed neurons as the primary cell type exhibiting enrichment (Figure 4B). Temporal specific expression analysis (SEA) indicated a significant developmental trajectory for the gene set, spanning from fetal stages into young adulthood (Figure 4C). Gene expression specificity was categorized using pSI threshold of 0.05 (Xu et al., 2014).

### PPI networks and hub genes

**Figure 5:**
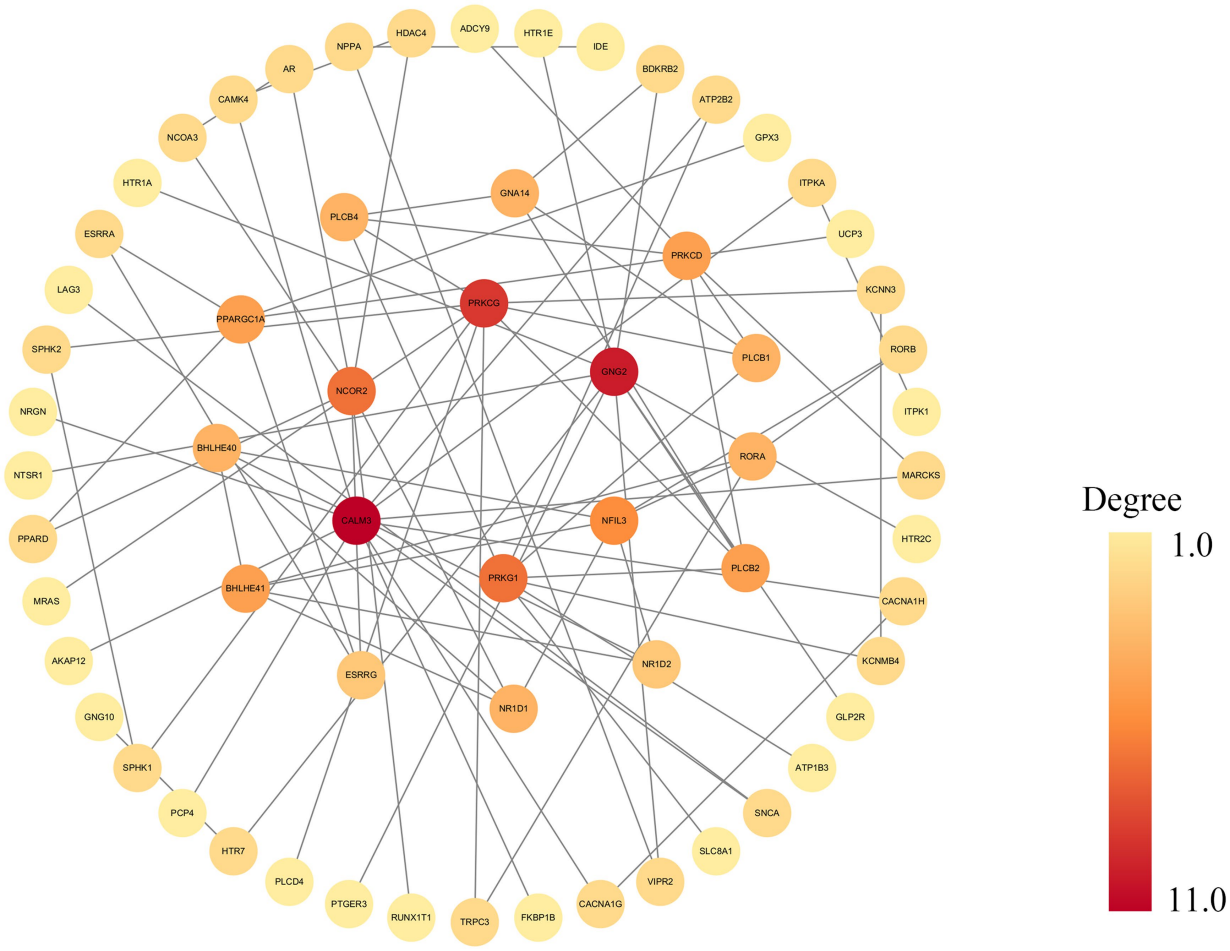
The networks from the STRING database are displayed in the main Cytoscape window. This figure illustrates a PPI network constructed from the STRING database and visualized using Cytoscape, generated with a high-confidence interaction score cutoff of 0.9. Nodes represent genes, and gray edges indicate functional or physical interactions. Node color reflects the degree of connectivity, ranging from yellow (low degree) to dark red (high degree). PPI, protein–protein interaction.

To identify key regulatory genes and their interaction landscape, a statistically significant PPI network was constructed from the differentially expressed genes, comprising nodes and edges. The network’s visualization in Cytoscape (Figure 5) demonstrates a clear core-periphery architecture, which is strong evidence of its non-random organization. Topological analysis using degree centrality revealed a densely interconnected central module of 10 hub genes. Among these, CALM3 and GNG2 emerged as the most prominent hubs, distinguished by their central position and highest number of interactions. They were closely associated with a second tier of key hubs, including PRKCG, PRKG1, NCOR2, NFIL3, NR1D1, NR1D2, BHLHE40, and BHLHE41. Collectively, these 10 genes form the functional core of the network, interacting extensively with each other and with numerous peripheral proteins.

### Neurotransmitters associated with neural correlates of JME

**Figure 6:**
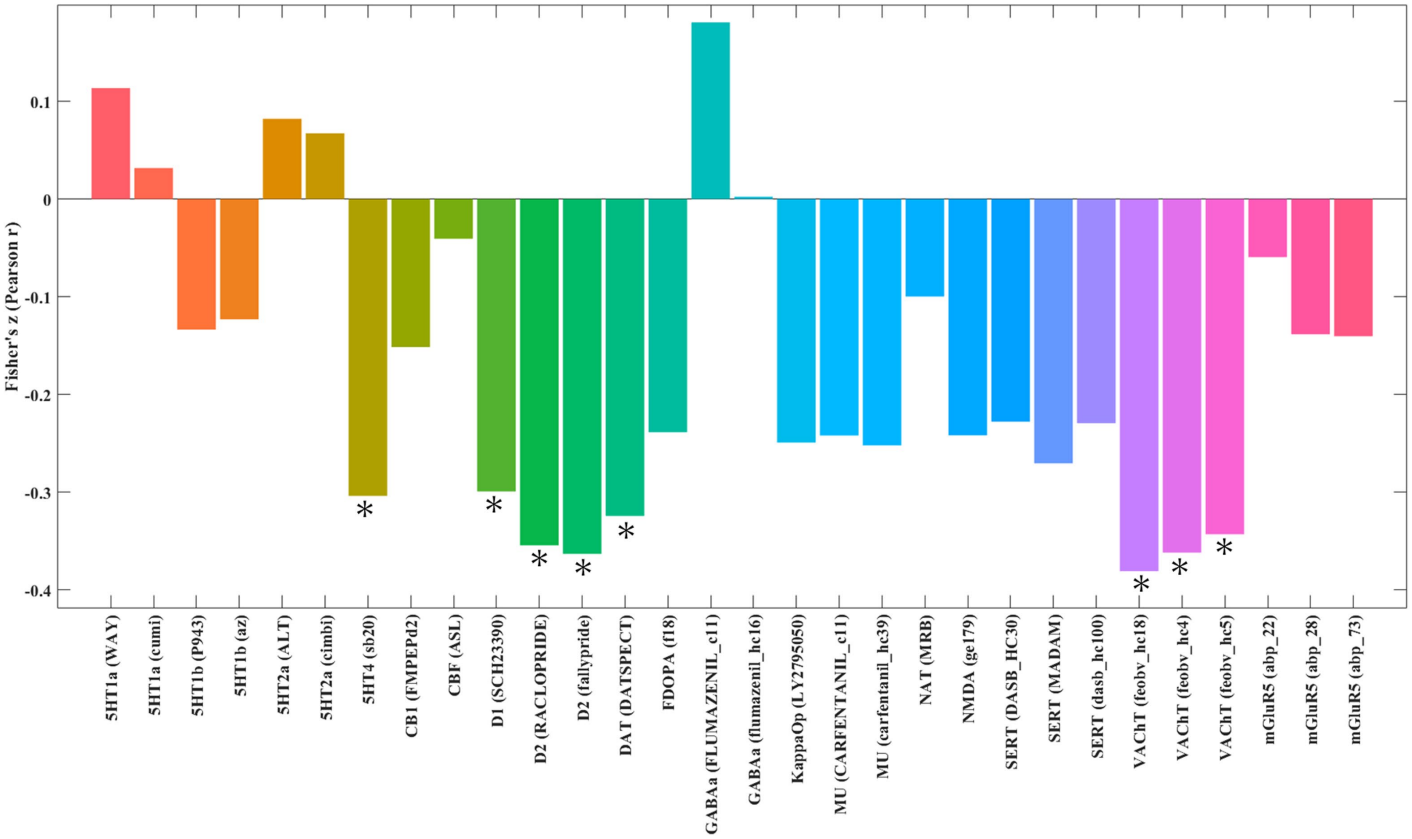
Spatial correlation between gray matter alterations in patients with juvenile myoclonic epilepsy and neurotransmitter system maps. The figure displays brain regions where the statistical map of gray matter alterations in juvenile myoclonic epilepsy shows significant spatial correlation with predefined maps of neurotransmitter distributions. *Indicates significant correlation at *p* < 0.01, *Indicates significant correlation at *p* < 0.01, corrected for multiple comparisons using the FDR method. 5HT1a, serotonin 1A receptor; 5HT1b, serotonin 1B receptor; 5HT2a, serotonin 2A receptor; 5HT4, serotonin 4 receptor; CB1, cannabinoid receptor 1; CBF (ASL), cerebral blood flow (arterial spin labeling); D1, dopamine receptor D1; D2, dopamine receptor D2; DAT, dopamine transporter; FDOPA, fluorodopa; FDR, false discovery rate; GABAa, gamma-aminobutyric acid A receptor; KappaOp, kappa opioid receptor; MU, mu-opioid receptor; NAT, norepinephrine transporter; NMDA, N-methyl-D-aspartate receptor; SERT, serotonin transporter; VAChT, vesicular acetylcholine transporter; mGluR5, metabotropic glutamate receptor 5.

Spatial correlation analysis revealed that the pattern of GM alterations in patients with JME was negatively correlated with the distribution of serotonin 4 receptor (5HT4) receptors (*r* = −0.30, *p* = 5.0 × 10^−4^), dopamine D1 receptors (D1) (*r* = −0.35, *p* = 2.0 × 10^−3^), dopamine D2 receptors (D2) (*r* = −0.36, *p* = 2.0 × 10^−4^), dopamine transporter (DAT) (*r* = −0.32, *p* = 1.0 × 10^−3^) and vesicular acetylcholine transporter (VAChT) (*r* = −0.38, *p* = 2.0 × 10^−4^; Figure 6). Reinforcing these findings, the results obtained using Bonferroni correction showed a high degree of overlap with those corrected by FDR, as detailed in the Supplementary Table S6.

## Discussion

By uniquely integrating a CBMA of brain structures with transcriptomic and neurotransmitter system analyses, this study provides a multi-level biological characterization of regional GM alterations in JME. Our updated CBMA first identified a distributed pattern of GM changes, with decreases in the left putamen, left postcentral gyrus, right precentral gyrus, and left inferior frontal gyrus, alongside increases in the right gyrus rectus, right parahippocampal gyrus, and left precuneus. Crucially, we then demonstrated that this structural pattern is not random; it is spatially correlated with a distinct gene expression profile enriched for key neuronal functions such as ion channel activity, synaptic processes, and cellular metabolism. These JME-associated genes were predominantly expressed in neurons and pituitary gland, with expression peaking during developmental periods relevant to JME onset. Finally, the spatial distribution of GM alterations was significantly correlated with the serotonin, dopamine, and acetylcholine neurotransmitter systems. Taken together, these convergent findings provide the first integrated model linking macroscopic brain structure in patients with JME to its underlying genetic and neurochemical architecture.

### GM alterations in JME

Building on previous work, our study provides an updated CBMA of GM alterations in JME by leveraging a larger cohort of studies. Our results both overlap with and diverge from the earlier CBMA by Kazis et al. (2021), offering a refined neuroanatomical map of the disorder. While we did not replicate findings like the thalamic atrophy, our analysis newly identified GM increases in the right gyrus rectus, right parahippocampal gyrus, and left precuneus, alongside GM decreases in the left putamen, left postcentral gyrus, right precentral gyrus, and left inferior frontal gyrus. These differences likely stem from the enhanced statistical power of our analysis, providing a more robust representation of structural signature.

The gyrus rectus, a component of the orbitofrontal cortex, is implicated in behavioral regulation, particularly regarding the processing of visceral and motor system information (Nestor et al., 2013; Kringelbach, 2005). Previous studies have reported gyrus rectus enlargement and metabolic abnormalities in epilepsy, suggesting its involvement in epileptic activity (Zhu et al., 2021; Xu et al., 2021). Similarly, the parahippocampal gyrus plays a critical role in memory and visuospatial functions (Lin et al., 2021). Alterations in the parahippocampal gyrus have been observed in JME, characterized by bilateral hypoperfusion and right-sided structural changes such as increased GM volume accompanied by decreased cortical thickness (CTh) (Sun et al., 2023; Anderson and Hamandi, 2011). We observed increased GM in the precuneus, a region previously implicated in JME (Kim et al., 2022) and known for its crucial role in complex cognition, including memory and self-processing (Dadario and Sughrue, 2023). This finding is consistent with functional MRI and electroencephalography studies demonstrating enhanced connectivity within this region in JME (van de Velden et al., 2023). Conversely, regions exhibiting GM reduction comprised the left putamen, left postcentral gyrus, right precentral gyrus, and left inferior frontal gyrus. As a dopamine-rich component of the basal ganglia, the putamen is central to motor control and executive cognitive functions (Ghandili and Munakomi, 2025; Luo et al., 2019), and its dysfunction is a common feature in various neuropsychiatric conditions (Keller et al., 2011; Kim et al., 2018). Our finding of reduced putamen volume is consistent with previous MRI and diffusion tensor imaging studies reporting volume and microstructural deficits in this region in patients with JME (Kim et al., 2018). The postcentral gyrus, part of the primary somatosensory cortex, functions to integrate sensory and motor information (Kropf et al., 2019). A convergence of evidence from electroencephalography, magnetoencephalography, and combined structural/functional MRI studies has implicated the postcentral gyrus in the pathophysiology of JME, linking its alterations to seizure modulation (Zhang et al., 2022; Routley et al., 2020; Sanjari Moghaddam et al., 2022). The inferior frontal gyrus is critically involved in various cognitive processes (Wang et al., 2020; Briggs et al., 2019). Reduced inferior frontal gyrus volume/perfusion in JME has been previously demonstrated through CTh and SPECT analyses (Joo et al., 2006; Pillai et al., 2017). Additionally, the precentral gyrus, essential for voluntary motor control, exhibited decreased GM, echoing prior volumetric and CTh findings in patients with JME (Zhang et al., 2022; Banker and Tadi, 2024; Lee and Park, 2019; Tae et al., 2008). These regional GM alterations span motor control, sensory processing, memory, and cognition, highlighting the extensive neural involvement in JME. Consistent with previous multimodal imaging studies, these findings help refine the definition of the disorder’s neuroanatomical substrate.

### Gene functional enrichment

Our analysis identified 926 genes whose spatial expression correlated with regional GM alterations in JME, revealing their significant enrichment in biological pathways central to epilepsy pathophysiology, such as ion channel activity, synaptic function, neuronal processes, and cellular metabolism. The enrichment for ion channel pathways directly aligns with the established genetic basis of JME (Smith and Walsh, 2020; Moody and Bosma, 2005). Mutations in ion channel genes have been established to alter neuronal excitability and increase seizure susceptibility (Yacoub et al., 2024). Consequently, ion channels are considered pivotal mediators in the pathophysiology of epilepsy and represent highly promising therapeutic targets (Zifkin et al., 2005). In addition, synaptic function was highly enriched. Balanced synaptic activity is essential for neural communication and synaptic plasticity (Pereda, 2014; Anderson et al., 2018). In JME, however, this homeostasis is compromised by synaptic abnormalities—including altered pruning and increased hyperconnectivity—which are thought to underlie the development of hyperexcitable networks (Craiu, 2013; Strigaro et al., 2015). Key features of JME include thalamocortical hyperexcitability, often driven by genetic factors affecting neuronal excitability (Assenza et al., 2020). Disruptions in calcium-dependent synaptic transmission and plasticity (Dolphin and Lee, 2020) further enhance this network hyperexcitability (Katano et al., 2012; Yacoub et al., 2024). Specifically, dysfunction of CACNA1E, often secondary to EFHC1 mutations, has been implicated in JME pathogenesis (Delgado-Escueta, 2007; Suzuki et al., 2004; D'Onofrio et al., 2024).

Developmental disturbances in neuronal processes, particularly abnormal calcium signaling that impairs circuit refinement (Wong and Ghosh, 2002), may contribute to the formation of dysfunctional cortico-thalamic circuits and elevated JME risk (Galli et al., 2018; Van Luijtelaar and Sitnikova, 2006). A prevailing mechanism is likely an imbalance in synaptic strength favoring excitation, heightening seizure susceptibility (Van Oostrum and Schuman, 2024).

While JME is not primarily classified as a metabolic disorder (Tumiene et al., 2022), our findings underscore the critical link between metabolic stability and neuronal health (Shetty et al., 2012; Gomez-Pinilla and Tyagi, 2013). The high metabolic demands during seizures, coupled with potential mitochondrial vulnerabilities (Bazzigaluppi et al., 2017; Zsurka and Kunz, 2015), may either intensify neuronal hyperexcitability or impair neuronal recovery. Metabolic dysfunction can exacerbate neuronal damage during seizures (Rho and Boison, 2022) and may play a broader role in the etiology of epilepsy. Genetic defects affecting cellular metabolism can disrupt brain development and neuronal excitability, ultimately raising epilepsy risk (Rho and Boison, 2022).

### TSEA, CSEA, SEA, PPI networks and hub genes

Further analyses using TSEA, CSEA, SEA, and PPI networks provided critical context for these genetic associations. The gene set identified in our study was strongly associated with JME and showed predominant expression in brain tissue, underscoring the neurological relevance of our findings. Moreover, TSEA revealed significant enrichment in the pituitary gland, introducing a potentially novel aspect to JME pathophysiology. Epilepsy and its treatments are known to disrupt pituitary function and cause endocrine disorders, highlighting the importance of clinical monitoring for endocrine health in patients with epilepsy (Li et al., 2024; Xiaotian et al., 2013). Given the pivotal role of hormonal modulation in neuronal excitability (Jamieson and Piet, 2022) and the clinical overlap between epilepsy and endocrine dysfunction (Verrotti et al., 2012; Marcovecchio et al., 2015), pituitary-expressed genes may underpin JME pathogenesis by shaping neurodevelopment or directly modulating seizure thresholds.

In agreement with these tissue-specificity findings, CSEA indicated that neurons are the primary cell type expressing these genes. This finding aligns with the neural substrates of JME and highlights the neuronal origin of GM alterations observed in patients with JME. SEA identified a critical neurodevelopmental window for gene expression in the cortex and thalamus, ranging from early/mid-fetal stages to young adulthood (Chaudhury et al., 2016; Jiang and Nardelli, 2016; Subramanian et al., 2019; Kolk and Rakic, 2022). This developmental timing aligns with important processes of brain maturation and parallels the typical adolescent onset of JME (Fayad et al., 2024; Genton et al., 2013; Koepp et al., 2014), supporting the idea that genetic disturbances during this window may have lasting impacts on brain structure and JME risk.

PPI network analysis identified central hub genes belonging to two key functional classes. The first class comprises transcriptional regulators integral to circadian rhythms and neurodevelopment, namely NFIL3, BHLHE41, BHLHE40, NR1D1, NR1D2, and NCOR2 (Yetkin et al., 2024; Koike et al., 2023; Hofstra and de Weerd, 2009; Zhang-Sun et al., 2023; Aninye et al., 2014). Although JME is clinically established as a sleep-related syndrome characterized by seizures shortly after awakening (Xu et al., 2018), the mechanistic role of circadian dysregulation remains controversial. We propose that these circadian hub genes provide a critical bridge, potentially integrating genetic background with environmental triggers like the sleep–wake cycle to modulate seizure susceptibility. The second class consists of key molecules in cellular signal transduction: CALM3, GNG2, PRKCG, and PRKG1. Notably, mutations in CALM3 and GNG2 are established monogenic causes of epilepsy (Lin et al., 2024; Helbig et al., 2014; Li et al., 2020). Notably, GNG2 is functionally critical in regulating epileptogenesis, as evidenced by the established pathogenicity of its obligate partner, GNB1, in epileptic encephalopathies (Petrovski et al., 2016). Consistent with this, GNG2 downregulation in epileptogenic tissues suggests a disruption in G-protein signaling (Li et al., 2020). Similarly, CALM3 has been characterized as a key modulator of neuronal inhibition mediated by the cAMP-PKA pathway (Li et al., 2020). Collectively, these findings suggest that dysfunction within these fundamental signaling networks may lower the seizure threshold, thereby contributing to JME susceptibility. Furthermore, PRKCG and PRKG1 regulate neuronal excitability (Guo et al., 2017; Struk et al., 2019), and are implicated in synaptic plasticity (Feil et al., 2009; Huang et al., 2025). This suggests that the dysfunction of the multiple hub genes we identified—from the upstream sensors CALM3 and GNG2 to the downstream kinases PRKCG and PRKG1—may ultimately converge on the impairment of synaptic function. Such disruption of synaptic transmission and plasticity, driven by molecular deficits, could be a key biological basis for the network-level hypersynchrony and seizure susceptibility seen in JME. The co-identification of hub genes from both transcriptional control and immediate signaling pathways suggests a composite pathogenic mechanism, involving both long-term dysregulation of gene expression and acute defects in neuronal signaling.

### Neurotransmitters associated with neural correlates of JME

Beyond its genetic underpinnings, our findings reveal that the brain structural abnormalities in patients with JME are also significantly tied to the neurochemical architecture. The pattern of regional GM alterations in JME exhibited significant correlations with the spatial distributions of several key neurotransmitter systems, notably involving serotonin (via 5-HT4 receptors), dopamine (via D1receptors, D2 receptors, and DAT), and acetylcholine (via VAChT).

Our analysis highlights a significant association with the serotonin system, specifically via the 5-HT4 receptor. This aligns with the well-established role of serotonin in regulating mood, anxiety, and sensorimotor functions (Ren et al., 2018; Jenkins et al., 2016)—all relevant to JME (Esmail et al., 2015; Meschaks et al., 2005; Bagdy et al., 2007). The deep involvement of the 5-HT system in JME is supported by extensive evidence, including the association of 5-HT transporter gene polymorphisms with the disorder (Esmail et al., 2015; Schenkel et al., 2011; Kauffman et al., 2009). Crucially, the clinical efficacy of serotonergic modulators like fenfluramine as an anti-seizure treatment provides support to this link (Bagdy et al., 2007; Sourbron and Lagae, 2022), although the specific therapeutic potential of targeting 5-HT4 receptors requires further investigation. We also identified significant correlations with the dopamine system and the acetylcholine system. The link to dopamine is particularly relevant given its crucial role in motor control and cognition (Robinson and Gradinaru, 2018), functions that are often impaired in JME. Dopaminergic dysregulation may contribute not only to seizure generation but also to the cognitive deficits associated with the condition (Ciumas et al., 2008; Landvogt et al., 2010). While this suggests a potential area for investigation, the direct therapeutic value of targeting the dopaminergic system in JME remains to be established. Similarly, the involvement of acetylcholine, a key modulator of learning and memory (Hasselmo, 2006), points to another layer of vulnerability. While translational applications remain to be fully realized, the spatial concordance observed here aligns with the fundamental contribution of cholinergic receptors to seizure susceptibility (Hogg et al., 2003), suggesting an intrinsic role in JME pathophysiology.

Collectively, these findings highlight the important contributions of serotonergic, dopaminergic, and cholinergic systems to the structural brain changes observed in JME, reinforcing the concept of neurotransmitter imbalance in the pathophysiology and thereby necessitating the translation of these insights into viable therapeutic strategies.

### Limitations

Several limitations should be acknowledged when interpreting the findings of this study. Firstly, consistent with standard CBMA methodologies, this study relied upon reported peak coordinates from the included publications rather than original statistical maps, which may inherently limit the spatial precision of the results (Radua et al., 2012). Secondly, methodological heterogeneity across studies (e.g., MRI field strengths, analysis pipelines) is an inherent limitation that may introduce potential variability in spatial localization. However, this variability underscores the necessity of our CBMA approach, which is designed to distill a robust consensus by identifying spatially consistent effects across technically diverse datasets (Radua and Mataix-Cols, 2012). Furthermore, clinical heterogeneity, particularly the widespread use of anti-seizure medications, is a significant confound. Insufficient granular data in the source studies (e.g., disease duration, specific subtypes) precluded meta-regression to disentangle these effects. Our findings therefore likely represent the combined effects of the disease and medication, underscoring the need for future studies on newly diagnosed, untreated patients. Thirdly, our reliance on normative atlases (AHBA and neurotransmitter maps) derived from healthy adult donors represents a significant constraint, creating a temporal and physiological mismatch that cannot capture JME-specific molecular alterations (e.g., those driven by seizures or medication). We acknowledge that ideal data would originate from matched patients with JME, but such large-scale, high-resolution resources are currently unavailable. However, our approach is valid within its hypothesis-generating objective. Operating on the assumption that the brain’s relative molecular spatial patterns are largely conserved, we correlated this canonical architecture with JME-related structural damage to identify systems implicated in regional vulnerability. Consequently, the observed associations should be rigorously interpreted not as direct evidence of molecular pathology, but as data-driven hypotheses whose primary value is to prioritize targets for future, patient-specific validation (e.g., via PET or transcriptomics). Finally, the study’s design is inherently correlational. Therefore, definitive causal relationships between the observed GM alterations, associated gene expression patterns, neurotransmitter system distributions, and the underlying pathophysiology of JME cannot be inferred solely from these data alone. Longitudinal studies and experimental validations are necessary to establish causal links.

## Conclusion

In summary, this study delineates robust and spatially consistent patterns of GM alterations in JME. Our updated CBMA demonstrated convergent GM reductions in regions central to sensorimotor control—including the left putamen, left postcentral gyrus, right precentral gyrus, and left inferior frontal gyrus—as well as GM increases in areas associated with emotion, memory, and self-referential processing, such as the right gyrus rectus, right parahippocampal gyrus, and left precuneus. Importantly, by integrating brain-wide gene expression and neurotransmitter receptor mapping, we provide the first comprehensive evidence linking these structural abnormalities to specific transcriptional and neurochemical signatures. Taken together, these findings offer novel insights into the multifactorial pathophysiology of JME, underscoring the intricate interplay between brain structure, genetic architecture, and neurochemical systems in this prevalent epilepsy syndrome. These hypothesis-generating findings offer a roadmap for future mechanistic studies and should be interpreted within the context of their reliance on normative human brain atlases.

## Data Availability

The original contributions presented in the study are included in the article/Supplementary material, further inquiries can be directed to the corresponding author/s.

## Footnotes

1
https://www.sdmproject.com/utilities/?show=Statistics

2
www.sdmproject.com

3
http://www.sdmproject.com/software/tutorial.pdf

4
https://www.nitrc.org/projects/mricron/

5
http://www.brain-map.org

6
http://geneontology.org/

7
https://github.com/murraylab/brainsmash

8
http://doughertytools.wustl.edu/TSEAtool.html

9
http://doughertytools.wustl.edu/CSEAtool.html

10
https://string-db.org/

11
https://github.com/juryxy/JuSpace

## Glossary

5HT4: Serotonin 4 receptor
AES-SDM: Anisotropic effect size version of signed differential mapping
AHBA: Allen Human Brain Atlas
CBMA: Coordinate-based meta-analysis
CSEA: Cell type specific expression analysis
CTh: Cortical thickness
D1: Dopamine receptor D1
D2: Dopamine receptor D2
DAT: Dopamine transporter
DS: Differential stability
FDR: False discovery rate
FSL: FMRIB software library
FWHM: Full width at half maximum
GCEA: Gene category enrichment analysis
GM: Gray matter
GO: gene ontology
HCs: Healthy controls
JME: Juvenile myoclonic epilepsy
MNI: Montreal Neurological Institute
MRI: Magnetic resonance imaging
PET: Positron emission tomography
PPI: Protein–protein interaction
pSI: Specificity index probability
RNA-seq: RNA sequencing
SEA: Specific expression analysis
SPECT: Single-photon emission computed tomography
SPM: Statistical parametric mapping
TSEA: Tissue specific expression analysis
VAChT: Vesicular acetylcholine transporter
VBM: Voxel-based morphometry
