# Supplementary material for: Transcriptional and neurotransmitter signatures associated with regional gray matter alterations in juvenile myoclonic epilepsy
Source: Front Mol Neurosci. 2026 Jan 29;19:1693722. doi: 10.3389/fnmol.2026.1693722 (PMC12894257; doi:10.3389/fnmol.2026.1693722)
Supplement: Supplementary file 1 [file Data_Sheet_1.docx]

**Table S1. Demographic information of the six adult donors in the AHBA**

| **Donor ID** | **Gender** | **Age (years)** | **Ethnicity** | **Hemispheres** |
| --- | --- | --- | --- | --- |
| H0351.1009 | Male | 57 | Caucasian | Left |
| H0351.1012 | Male | 31 | Caucasian | Left |
| H0351.1015 | Female | 49 | Hispanic | Left |
| H0351.1016 | Male | 55 | Caucasian | Left |
| H0351.2001 | Male | 24 | African American | Both |
| H0351.2002 | Male | 39 | African American | Both |

Abbreviations: AHBA, Allen Human Brain Atlas.
