# Supplementary material for: Transcriptional and neurotransmitter signatures associated with regional gray matter alterations in juvenile myoclonic epilepsy
Source: Front Mol Neurosci. 2026 Jan 29;19:1693722. doi: 10.3389/fnmol.2026.1693722 (PMC12894257; doi:10.3389/fnmol.2026.1693722)
Supplement: Supplementary file 2 [file Data_Sheet_2.docx]

**Table S2. Sample and imaging characteristics of the studies included in the CBMA**

| **Study** | **Subjects (females)** | | **Mean age (SD)** | | **Illness duration (months)** | **MRI field strength** | **Software** | **Threshold** |
| --- | --- | --- | --- | --- | --- | --- | --- | --- |
|  | **JME** | **HCs** | **JME** | **HCs** |  |  |  |  |
| Woermann et al., (1999) ^1^ | 20 (12) | 30 (16) | 25 (NA) | 27 (NA) | NA | 1.5 T | SPM96 | FDR (*p* < 0.05) |
| Betting et al., (2006) ^2^ | 44 (30) | 47 (23) | 32 (9.0) | 32 (14) | NA | 2.0 T | SPM2 | FDR (*p* < 0.01) |
| Woo et al., (2006) ^3^ | 19 (13) | 19 (13) | 22.6 (4.0) | 22.9 (7.3) | 7.9 (4.68) | 1.5 T | SPM2 | FDR (*p* < 0.05) |
| Kim et al., (2007) ^4^ | 25 (15) | 44 (27) | 22.7 (5.1) | 23.1 (4.3) | 6.1 (3.80) | 1.5 T | SPM2 | uncorr |
| Roebling et al., (2009) ^5^ | 19 (16) | 20 (16) | 24.2 (9.9) | 24.4 (9.2) | NA | 1.5 T | SPM2 | FDR (*p* < 0.05) |
| Ciumas et al., (2010) ^6^ | 13 (4) | 12 (12) | 37 (13.0) | 33 (11.0) | 22 (13.00) | 1.5 T | SPM2 | FDR (*p* < 0.05) |
| Liu et al., (2011) ^7^ | 15 (12) | 15 (12) | 21 (4.0) | 21 (4.0) | 15.1 (10.70) | 1.5 T | SPM8 | FDR (*p* < 0.05) |
| O’muircheartaigh et al., (2011) ^8^ | 28 (16) | 55 (27) | 33.61 (10.1) | 30.11 (7.7) | 20.24 (NA) | 3.0 T | SPM8 | FDR (*p* < 0.05) |
| Saini et al., (2013) ^9^ | 40 (19) | 19 (6) | 22.8 (5.3) | 24.5 (4.2) | 7.2 (5.17) | 3.0 T | SPM5 | FDR (*p* < 0.05) |
| Swartz et al. (2016) ^10^ | 17 (10) | 17 (10) | 33.8 (10.4) | 35.4 (11.7) | NA | 1.5 T | SPM8 | NA |
| Knake et al., (2017) ^11^ | 20 (12) | 20 (12) | 34.8 (13.31) | 33.50 (13.57) | 20.42 (14.90) | 3.0 T | FSL | Alphasim (*p* < 0.01) |
| Zhong et al., (2018) ^12^ | 25 (17) | 24 (15) | 24.68 (6.4) | 25.41 (7.7) | 12.16 (7.40) | 3.0 T | SPM8 | FDR (*p* < 0.05) |
| Ozturk et al., (2020) ^13^ | 15 (9) | 43 (24) | 16.1 (3.2) | 15.9 (2.8) | NA | 1.5 T | SPM8 | NA |
| Zhang et al., (2022) ^14^ | 67 (38) | 56 (36) | 23.07 (5.89) | 26.77 (4.68) | 8.67 (5.77) | 3.0 T | SPM12 | FDR (*p* < 0.05) |
| Ke et al., (2024) ^15^ | 27 (12) | 27 (15) | 17.5 (4.9) | 19.6 (4.7) | 40.4 (50.00) | 3.0 T | SPM12 | NA |

Abbreviations: CBMA, coordinate-based meta-analysis; FDR, false discovery rate; FSL, FMRIB software library; HCs, healthy controls; JME, juvenile myoclonic epilepsy; NA, not available; SD, standard deviation; Uncorr, uncorrected.

**References**

(1) Woermann, F. G.; Free, S. L.; Koepp, M. J.; Sisodiya, S. M.; Duncan, J. S. Abnormal cerebral structure in juvenile myoclonic epilepsy demonstrated with voxel-based analysis of MRI. *Brain* **1999**, *122 ( Pt 11)*, 2101-2108. DOI: 10.1093/brain/122.11.2101 From NLM.

(2) Betting, L. E.; Mory, S. B.; Li, L. M.; Lopes-Cendes, I.; Guerreiro, M. M.; Guerreiro, C. A.; Cendes, F. Voxel-based morphometry in patients with idiopathic generalized epilepsies. *Neuroimage* **2006**, *32* (2), 498-502. DOI: 10.1016/j.neuroimage.2006.04.174 From NLM.

(3) Woo, S. T.; Seung, B. H.; Eun, Y. J.; Sun, J. H.; Cho, J. W.; Dae, W. S.; Lee, J. M.; In, Y. K.; Hong, S. B.; Kim, S. I. Structural brain abnormalities in juvenile myoclonic epilepsy patients: Volumetry and voxel-based morphometry. *Korean Journal of Radiology* **2006**, *7* (3), 162-172, Article. DOI: 10.3348/kjr.2006.7.3.162 Medline.

(4) Kim, J. H.; Lee, J. K.; Koh, S. B.; Lee, S. A.; Lee, J. M.; Kim, S. I.; Kang, J. K. Regional grey matter abnormalities in juvenile myoclonic epilepsy: a voxel-based morphometry study. *Neuroimage* **2007**, *37* (4), 1132-1137. DOI: 10.1016/j.neuroimage.2007.06.025 From NLM.

(5) Roebling, R.; Scheerer, N.; Uttner, I.; Gruber, O.; Kraft, E.; Lerche, H. Evaluation of cognition, structural, and functional MRI in juvenile myoclonic epilepsy. *Epilepsia* **2009**, *50* (11), 2456-2465. DOI: 10.1111/j.1528-1167.2009.02127.x From NLM.

(6) Ciumas, C.; Wahlin, T.-B. R.; Espino, C.; Savic, I. The dopamine system in idiopathic generalized epilepsies: Identification of syndrome-related changes. *Neuroimage* **2010**, *51* (2), 606-615. DOI: 10.1016/j.neuroimage.2010.02.051.

(7) Liu, M.; Concha, L.; Beaulieu, C.; Gross, D. W. Distinct white matter abnormalities in different idiopathic generalized epilepsy syndromes. *Epilepsia* **2011**, *52* (12), 2267-2275. DOI: 10.1111/j.1528-1167.2011.03313.x From NLM.

(8) O'Muircheartaigh, J.; Vollmar, C.; Barker, G. J.; Kumari, V.; Symms, M. R.; Thompson, P.; Duncan, J. S.; Koepp, M. J.; Richardson, M. P. Focal structural changes and cognitive dysfunction in juvenile myoclonic epilepsy. *Neurology* **2011**, *76* (1), 34-40. DOI: 10.1212/WNL.0b013e318203e93d From NLM.

(9) Saini, J.; Sinha, S.; Bagepally, B. S.; Ramchandraiah, C. T.; Thennarasu, K.; Prasad, C.; Taly, A. B.; Satishchandra, P. Subcortical structural abnormalities in juvenile myoclonic epilepsy (JME): MR volumetry and vertex based analysis. *Seizure* **2013**, *22* (3), 230-235. DOI: 10.1016/j.seizure.2013.01.001 From NLM.

(10) Swartz, B. E.; Spitz, J.; Vu, A. L.; Mandelkern, M.; Su, M. L. Heterogeneity of anatomic regions by MR volumetry in juvenile myoclonic epilepsy. *Acta Neurol Scand* **2016**, *134* (4), 300-308. DOI: 10.1111/ane.12544 From NLM.

(11) Knake, S.; Roth, C.; Belke, M.; Sonntag, J.; Kniess, T.; Krach, S.; Jansen, A.; Sommer, J.; Paulus, F. M.; Carl, B.; et al. Microstructural white matter changes and their relation to neuropsychological deficits in patients with juvenile myoclonic epilepsy. *Epilepsy Behav* **2017**, *76*, 56-62. DOI: 10.1016/j.yebeh.2017.08.031 From NLM.

(12) Zhong, C.; Liu, R.; Luo, C.; Jiang, S.; Dong, L.; Peng, R.; Guo, F.; Wang, P. Altered Structural and Functional Connectivity of Juvenile Myoclonic Epilepsy: An fMRI Study. *Neural Plast* **2018**, *2018*, 7392187. DOI: 10.1155/2018/7392187 From NLM.

(13) Öztürk, Z.; Güneş, A.; Karalok, Z. S. Subcortical gray matter changes in pediatric patients with new-onset juvenile myoclonic epilepsy. *Epilepsy Behav* **2020**, *104* (Pt A), 106860. DOI: 10.1016/j.yebeh.2019.106860 From NLM.

(14) Zhang, J.; Wu, D.; Yang, H.; Lu, H.; Ji, Y.; Liu, H.; Zang, Z.; Lu, J.; Sun, W. Correlations Between Structural Brain Abnormalities, Cognition and Electroclinical Characteristics in Patients With Juvenile Myoclonic Epilepsy. *Front Neurol* **2022**, *13*, 883078. DOI: 10.3389/fneur.2022.883078 From NLM.

(15) Ke, M.; Hou, L.; Liu, G. The co-activation patterns of multiple brain regions in Juvenile Myoclonic Epilepsy. *Cogn Neurodyn* **2024**, *18* (2), 337-347. DOI: 10.1007/s11571-022-09838-7 From NLM.
