# Supplementary material for: Transcriptional and neurotransmitter signatures associated with regional gray matter alterations in juvenile myoclonic epilepsy
Source: Front Mol Neurosci. 2026 Jan 29;19:1693722. doi: 10.3389/fnmol.2026.1693722 (PMC12894257; doi:10.3389/fnmol.2026.1693722)
Supplement: Supplementary file 3 [file Data_Sheet_3.docx]

**Table S3 Enrichment results of the genes related to juvenile myoclonic epilepsy**

| **Category** | **Name** | **Category score** | ***P* value** | **Count** |
| --- | --- | --- | --- | --- |
| BP | protein heterooligomerization | -0.035507713 | 0.0043 | 13 |
| BP | positive regulation of interferon-beta production | -0.033898745 | 0.0225 | 10 |
| BP | regulation of striated muscle cell apoptotic process | -0.023359449 | 0.0247 | 21 |
| MF | protease binding | -0.024816681 | 0.0146 | 38 |
| MF | antigen binding | -0.060316607 | 0.0193 | 28 |
| BP | deoxyribonucleotide catabolic process | 0.027514416 | 0.0042 | 12 |
| BP | deoxyribose phosphate catabolic process | 0.027514416 | 0.0042 | 12 |
| BP | regulation of astrocyte differentiation | 0.040755699 | 0.0137 | 10 |
| BP | response to electrical stimulus | 0.022806455 | 0.0163 | 11 |
| BP | calcium-dependent cell-cell adhesion via plasma membrane cell adhesion molecules | 0.041735558 | 0.0245 | 15 |
| CC | postsynaptic specialization membrane | 0.035609346 | 0.0172 | 75 |
| CC | postsynaptic density membrane | 0.033948761 | 0.0236 | 58 |
| CC | axoneme | 0.023883421 | 0.0241 | 35 |

Abbreviations: BP, biological process; CC, cellular component; MF, molecular function.
