# Supplementary material for: Transcriptional and neurotransmitter signatures associated with regional gray matter alterations in juvenile myoclonic epilepsy
Source: Front Mol Neurosci. 2026 Jan 29;19:1693722. doi: 10.3389/fnmol.2026.1693722 (PMC12894257; doi:10.3389/fnmol.2026.1693722)
Supplement: Supplementary file 4 [file Data_Sheet_4.docx]

**Table S4. Gene categories associated with the neural correlates of JME at thresholds 40%**

| **Category** | **Name** | **Category score** | ***P* value** | **Count** |
| --- | --- | --- | --- | --- |
| MF | antigen binding | -0.06066 | 0.0202 | 25 |
| BP | icosanoid metabolic process | -0.05475 | 0.0155 | 17 |
| BP | leukocyte cell-cell adhesion | -0.04318 | 0.0104 | 18 |
| BP | regulation of alternative mRNA splicing, via spliceosome | 0.035115 | 0.0199 | 10 |

Abbreviations: BP, biological process; JME, juvenile myoclonic epilepsy; MF, molecular function.
