# Supplementary material for: Transcriptional and neurotransmitter signatures associated with regional gray matter alterations in juvenile myoclonic epilepsy
Source: Front Mol Neurosci. 2026 Jan 29;19:1693722. doi: 10.3389/fnmol.2026.1693722 (PMC12894257; doi:10.3389/fnmol.2026.1693722)
Supplement: Supplementary file 5 [file Data_Sheet_5.docx]

**Table S5. Gene categories associated with the neural correlates of JME at thresholds 60%**

| **Category** | **Name** | **Category score** | ***P* value** | **Count** |
| --- | --- | --- | --- | --- |
| MF | antigen binding | -0.056999324 | 0.0184 | 29 |
| BP | response to iron ion | -0.041256785 | 0.0111 | 12 |
| BP | icosanoid metabolic process | -0.031481312 | 0.0200 | 34 |
| BP | purine nucleoside monophosphate metabolic process | -0.029879895 | 0.0187 | 17 |
| BP | purine ribonucleoside monophosphate metabolic process | -0.029879895 | 0.0187 | 17 |
| BP | protein heterooligomerization | -0.029589319 | 0.0228 | 15 |
| MF | protease binding | -0.023392435 | 0.0056 | 40 |
| BP | regulation of DNA binding | -0.022450398 | 0.0218 | 21 |
| BP | deoxyribonucleotide catabolic process | 0.023226548 | 0.0117 | 13 |
| BP | deoxyribose phosphate catabolic process | 0.023226548 | 0.0117 | 13 |
| BP | pyrimidine-containing compound catabolic process | 0.026020574 | 0.0124 | 16 |
| BP | motor behavior | 0.040077153 | 0.0225 | 14 |
| MF | ligand-gated calcium channel activity | 0.041439960 | 0.0223 | 11 |

Abbreviations: BP, biological process; JME, juvenile myoclonic epilepsy; MF, molecular function.
