# Supplementary material for: Transcriptional and neurotransmitter signatures associated with regional gray matter alterations in juvenile myoclonic epilepsy
Source: Front Mol Neurosci. 2026 Jan 29;19:1693722. doi: 10.3389/fnmol.2026.1693722 (PMC12894257; doi:10.3389/fnmol.2026.1693722)
Supplement: Supplementary file 6 [file Data_Sheet_6.docx]

**Table S6 *P*-values of neurotransmitters after Bonferroni correction**

| Neurotransmitter | 5-HT4 | D2 | DAT | VAChT |
| --- | --- | --- | --- | --- |
| *p*-value | 1.8×10^-2^ | 5.8×10^-3^ | 3.6 × 10⁻² | 6.0×10^-3^ |

Abbreviations: 5HT4, serotonin 4 receptor; D2, dopamine receptor D2; DAT, dopamine transporter; VAChT, vesicular acetylcholine transporter.
