# Supplementary figures and images for: Transcriptional and neurotransmitter signatures associated with regional gray matter alterations in juvenile myoclonic epilepsy
Source: Front Mol Neurosci. 2026 Jan 29;19:1693722. doi: 10.3389/fnmol.2026.1693722 (PMC12894257; doi:10.3389/fnmol.2026.1693722)

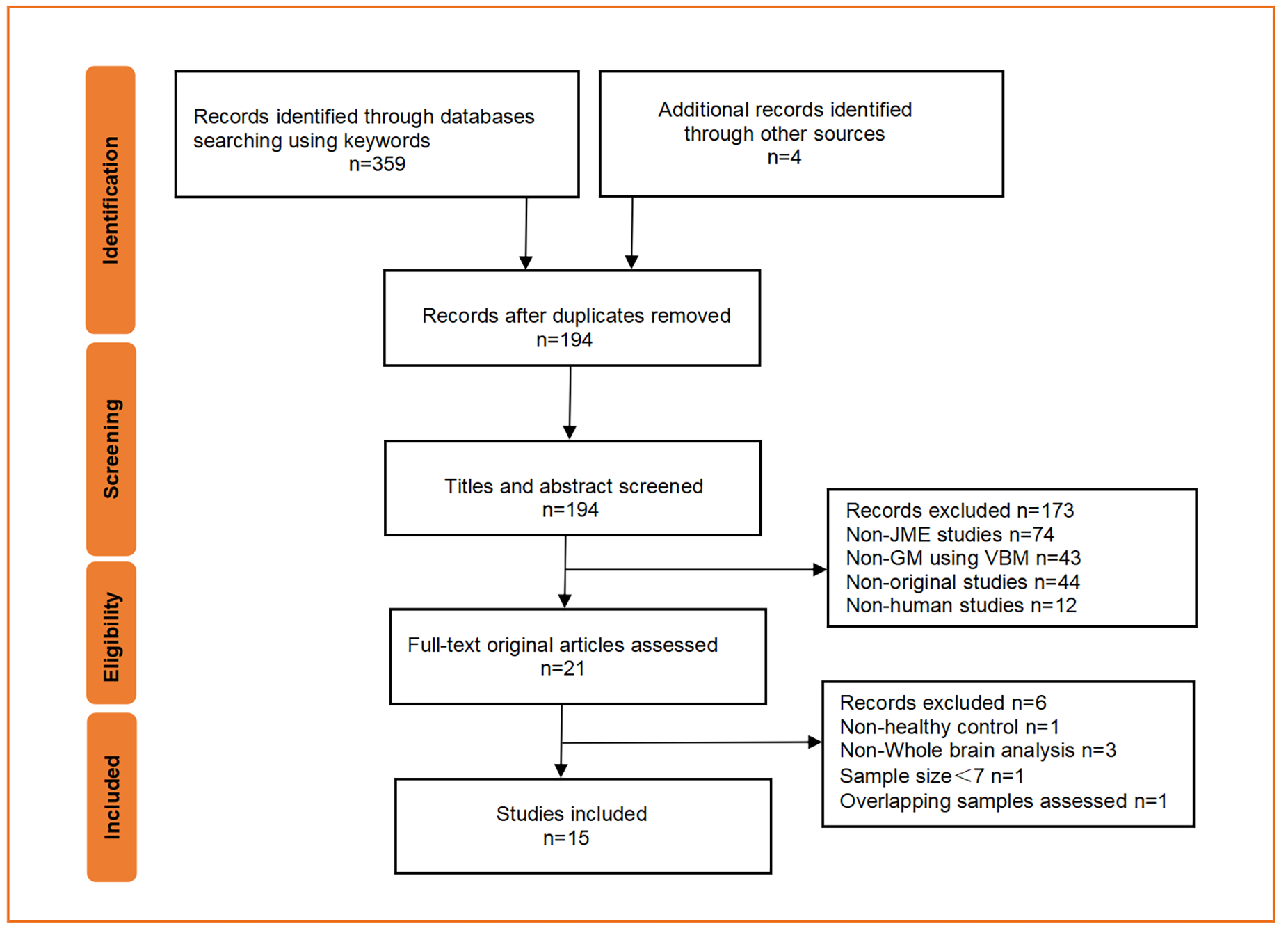

Supplement: Supplementary file 7 [file Image_1.tif]
